# Syndrome Evaluation System for Simultaneous Detection Pathogens Causing Acute Encephalitic Syndrome in India, Part-1: Development and Standardization of the Assay

**DOI:** 10.3389/fmed.2018.00208

**Published:** 2018-07-26

**Authors:** Sunil Govekar, Siddharth Anand, Latha P. Lakshman, Ravi Vasanthapuram, Ravikumar V. Banda

**Affiliations:** ^1^Research and Development, XCyton Diagnostics Limited, Bangalore, India; ^2^Department of Neurovirology, National Institute of Mental Health and Neurosciences, Bangalore, India

**Keywords:** acute encephalitis syndrome, simultaneous detection, molecular diagnostics, syndrome evaluation system, development

## Abstract

A large number of organisms are known to cause acute encephalitic syndrome (AES). A number of diagnostic tests have to be performed in order to arrive at a probable pathogen causing AES thus making it a very time consuming, laborious and expensive. The problem is further compounded by the lack of availability of sufficient volume of Cerebrospinal fluid (CSF). Thus, there is an urgent need of a diagnostic tool for the simultaneous detection of all probable pathogens responsible for causing AES. Here we report the development of a novel diagnostic method, Syndrome Evaluation System (SES) for the simultaneous detection of 22 pathogens including RNA and DNA Viruses, bacteria, fungi, and parasite all endemic to India and Southeast Asia in a single sample using a novel multiplexing strategy. Syndrome Evaluation System (SES) involves isolation of nucleic acid, multiplex amplification of the DNA, and cDNA followed by identification of the amplified product by sequence specific hybridization on SES platform with the final read out being a visually recordable colored signal. The total time required to carry out this diagnostic procedure is 7 h. The SES was standardized using the commercially available vaccines, panels and cell culture grown quantified viruses/bacteria/fungi. The limit of detection (LOD) of SES ranged between 0.1 and 50 viral particles per ml of CSF and 100 to 200 bacterial cells or 5 parasites per ml of CSF, along with 100% specificity. Precision studies carried out as per the Clinical Laboratory Improvement Amendments (CLIA) guidelines, using two concentrations of each pathogen one the LOD and the other double the LOD, clearly demonstrated, that inter/intra assay variability was within the limits prescribed by the guidelines. SES is a rapid molecular diagnostic tool for simultaneous identification of 22 etiological agents of AES encountered both in sporadic and outbreak settings.

## Introduction

Acute Encephalitic Syndrome (AES) is a major public health problem in several parts of Asia, especially India. A variety of pathogens are known to cause AES. They include DNA and RNA viruses, bacteria, fungi, and parasites (1). Despite the availability of several modalities, the etiological diagnosis of AES remains a challenge. Amongst the methods available at present for diagnosis of AES, routine Cerebrospinal fluid (CSF) cell count, protein, and glucose estimation as well as smear examinations, virus culture, and immunological tests are neither sensitive nor specific enough to provide a precise etiological diagnosis (2, 3). Similarly, imaging techniques, though useful, only provides information pertaining to the anatomic site of infection in the Central Nervous System (CNS) and does not provide specific etiological diagnosis. In addition, a major limitation in laboratory diagnosis is the availability of an appropriate specimen. Although CSF is the specimen of choice, it is most often not available in all cases of AES, and when available the volume quite often is insufficient for carrying out sequential diagnosis of a variety of pathogens that can cause CNS infections. Lastly, the diagnostic tools available at present are expensive, time consuming (4) and are not designed for simultaneous detection of all pathogens in a single assay.

Molecular diagnostic methods such as Polymerase chain reaction (PCR) have greatly facilitated the rapid identification of the etiological agent responsible for AES in many cases. In a large prospective study carried out at the John Radcliffe Hospital, Oxford, (5) had demonstrated the utility of a multiplex PCR, for the detection of viral infections of CNS. They further noted that an algorithmic approach minimized testing costs and enabled rapid identification of viral pathogens by PCR. This study underscored the advantage of using a multiplex approach for diagnosis of CNS infections. However, subsequent attempts by this group as well as other groups were restricted to the identification of etiological agents belonging to a certain family of viruses such as Enteroviridae (6–9) and Herpesviridae (10–13, 32). Consequently, methods for simultaneous detection of other agents of AES such as bacteria, parasites and fungi have not received any attention. Therefore, an attempt was made in this study to develop a comprehensive Syndrome Evaluation System (SES) for simultaneous detection of 22 common AES pathogens which included DNA and RNA viruses, bacteria, fungi and parasites.

## Materials and methods

### Source of pathogens and standards

The list of pathogens used in the development of the SES as well as their source is presented in Table 1. Standard strains of Japanese Encephalitis Virus (JEV), Dengue and Chikungunya virus, Challenge Virus Strain (CVS) of Rabies, Coxsackie B1-B6 viruses were available at the Department of Neurovirology, National Institute of Mental Health and Neuro Sciences (NIMHANS). All these viruses were propagated in standard cell lines obtained from National Center for Cell Sciences, Pune, India. Commercially available live attenuated viral vaccines such as *Varilrix* for Varicella zoster virus, *Tresivac*, for Measles and Rubella virus, *Oral Polio Vaccine* a representative of viruses belonging to the family of *Enteroviridae* and *Mumps vaccine* for Mumps virus were procured commercially and used as reference starins. Similarly, commercially available proficiency panels of for HSV, CMV, JC virus, HHV-6, and T. gondii were procured from Qnostics, UK and used in the study as standards. For Nipah and Chandipura viruses, the “N” gene plasmid constructs (obtained as kind gifts from scientists) were used as standards. ATCC strains of *S. pneumoniae, N. meningitidis, H. influenzae, C.neoformans*, and a standard strain of *M.tuberculosis* (H37Rv) available in the laboratory were used as standards. Standards used in the assay was quantified either by calculating TCID_50_/FFID_50_ for cell culture grown RNA viruses such as JEV, Chikungunya, and Rabies. Quantitation of bacterial cultures was carried out using McFarland standards. The TCID_50_ values provided by the manufacturers were used in case of all commercial vaccines such as *Varilrix, Tresivac, Mumps*, and *Oral Polio* were used for calculating the limit of detection of the assay.

**Table 1:** Source of pathogens used in the development of SES.

**Sl no**	**Name of the pathogen**	**Viral strains/plasmid**	**Source**
1	Herpes Simplex Virus	HSVDNA08	QCMD^*^
2	Cytomegalovirus	CMVDNA08	QCMD
3	Varicella Zoster Virus	Oka strain	Varilrix, GlaxoSmithkline
4	Human Herpes Virus-6	HHV6DNA09	QCMD
5	John Cunningham Virus	JCBKDNA10	QCMD
6	*S. pneumoniae*	6301	ATCC
7	*H. influenzae*	33533	ATCC
8	*N. meningitidis*	13077	ATCC
9	*M. tuberculosis*	H37Rv	National Tuberculosis Institute, Bangalore, India
10	*C. neoformans*	Clinical isolate	St. John's Hospital, Bangalore, Inida
11	*T. gondii*	TGDNA08	QCMD
12	JEV	Clinical isolate	National Institute of Virology, Pune, India
13	Dengue	Clinical isolate	National Institute of Virology, Pune, India
14	West Nile	Clinical isolate	National Institute of Virology, Pune, India
15	Chikungunya	Clinical isolate	Defense Research and Development Establishment, Gwalior, India
16	Rabies	CVS	Central Research Institute, Kasauli, India
17	Enteroviruses	Standard strain	Enterovirus Research Center, Mumbai, India
18	Measles	Edmonston-Zagreb	Tresivac, Serum Institute of India
19	Mumps	L-Zagreb	Tresivac, Serum Institute of India
20	Nipah	“N” gene plasmid	University of Malay, Malaysia
21	Rubella	Wistar RA 27/3	Tresivac, Serum Institute of India
22	Chandipura	“N” gene plasmid	University College of Science and Technology, Kolkotta, India

* *QCMD: Standards for HSV, CMV, JC and HHV-6 were obtained from QCMD, London, UK. The European Union supported Quality Control Concerted Action (QCCA) established a series of pilot molecular quality schemes in diagnostic virology & microbiology. The QCCA quality initiatives proved very successful and were supported by the European Society of Clinical Virology (ESCV) and the European Society for Clinical Microbiology & Infectious Diseases (ESCMID). QCMD is a non profit organization set up by EU under QCCA initiative*.

### Primer and probe design

Primers and probes used in the development of SES were designed using full length genome sequences or complete coding sequence obtained from Gene Bank of NCBI (Table 2). Care was taken to ensure that these were in the conserved regions of the genome to minimize variability in the assay and were synthesized commercially (Metabion Inc., Germany).

**Table 2:** Name of the pathogen, Gene targets chosen for primer design and the gene bank accession numbers.

**Sl. no**	**Pathogen**	**Gene target**	**Gene bank accession number**
1	Measles	“N” Gene	AB016162.1
2	Mumps	“N” Gene	AB040874.1
3	Rubella	“E1” Gene	M15240.1
4	Nipah	“N” Gene	AF212302.2
5	Rabies	“N” Gene	M13215.1
6	JEV	“NS3” Gene	AF27250
7	Chikungunya	“N” Gene	EF027139.1
8	Chandipura	“N” Gene	AY614731
9	Polio	5′ UTR	GQ984141.1
10	Dengue	PreM-E	AB609589
11	West Nile	“E” gene	KC601756.1
12	HSV	• Glycoprotein D •Untranslated region 44 •DNA polymerase	X14112
13	CMV	• Glycoprotein O •Untranslated region 83 •Morphological transformation region II	AY446894.2
14	VZV	• ORF 29 •DNA Polymerase	X04370
15	HHV-6	DNA Polymerase	X83413
16	JC	Small “t” protein	AB118232
17	Streptococcus pneumoniae	“LytA” Gene	CP003357.1
18	Neisseria meningitidis	“OpaA” Gene	CP002424.1
19	Haemophillus influenzae	“Lic” Gene	FQ670204.1
20	Mycobacterium tuberculosis	“MPB 64”	AL123456.3
21	Toxoplasma gondii	“B1” Gene	AF179871.1
22	Cryptococcus neoformans	18S rRNA	GQ850137.1

### Assay development

#### Nucleic acid extraction

Nucleic acid was extracted from the standard strains using commercial columns (Qiagen, USA) as per the procedure specified in the instruction manual provided by the manufacturer. The extraction kit used for RNA viruses was the Qiagen QIAamp Viral RNA Mini kit (Cat No. 52906) while that used for DNA organisms was the Qiagen QIAamp DNA mini kit (Cat no: 51306).

#### cDNA synthesis and nucleic acid amplification

Reverse transcription of total RNA extracted from the viral standards were carried out using a commercial cDNA Archive Kit (ABI, USA). cDNA was synthesized at 45°C for 30 min in a final volume of 50 μl, using 100 nM pathogen specific primers, 25 μl of RNA, 2 μl Multi-Scribe reverse transcriptase (50 Units/μl), 2 μl of 25 × dNTP mix, 5 μl of 10 × RT buffer and 1μl of RNAse inhibitor.

Nucleic acid amplification was standardized in a 50 μl volume containing 4 mM magnesium chloride, 0.2 mM deoxynucleoside triphosphates, 50 to 300 nM concentration of each primer set and 1U of Taq polymerase (ABI, USA). The initial denaturation step was carried out at 95°C for 10 min followed by 40 cycles of denaturation at 95°C for 45 s, annealing at 60°C for 45 s and extension at 72°C for 45 s in a thermal cycler (*Bio-Rad*, UK). The amplification products were detected by electrophoresis on a 4% agarose gel and/or hybridization on the SES platform.

#### Hybridization

**Figure 1:**
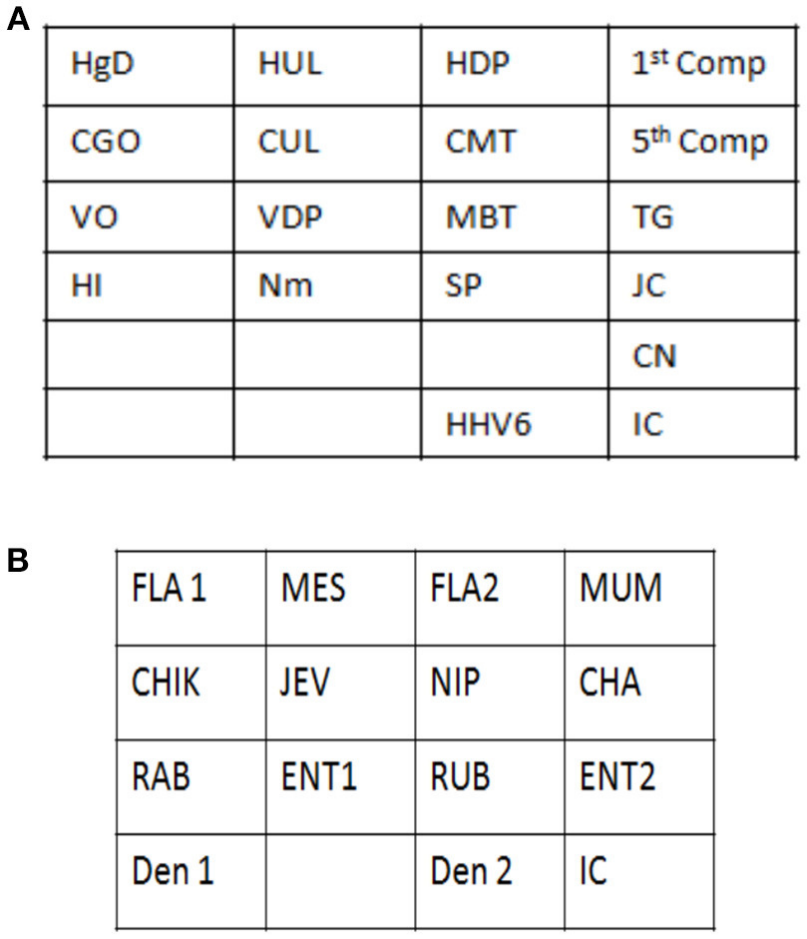
**(A)** Template depicting the position of various probes used for detection of DNA pathogens on the Syndrome Evaluation System platform. Row 1:HgD = Glycoprotein D of HSV 1 and 2; HUL = UL 44 of HSV 1; HDP = DNA polymerase of HSV 1 and 2; 1st Comp = complementary strand of HgD gene of HSV 1 and 2; Row 2: CGO = Glycoprotein O of CMV; CUL = UL 63 of CMV; CMT = Morphological transformation Region of CMV; 5th Comp = complementary strand of CMT gene of CMV; Row 3: VO = ORF 29 gene of VZV; VDP = DNA polymerase gene of VZV; MBT = M. tuberculosis; TG = T.gondii; Row4: HI = H. influenzae; Nm = N. meningitidis; SP = S. Pneumoniae and JC = JC virus; Row 5: CN = C. neoformans; Row 6: HHV-6 = Human Herpes Vvirus-6; IC = Internal control. **(B)** Template depicting the position of various probes used for detection of RNA pathogens on the Syndrome Evaluation System platform. Row 1: FLA1 = Flavivirus; MES = Measles; FLA2 = Complimentary strand of FLA1 gene of Flavivirus; MUM = Mumps; Row 2: CHIK = Chikungunya; JEV = Japanese encephalitis virus; NIP = Nipah; CHA = Chandipura; Row 3: RAB = Rabies; ENT1 = Enteroviruses; RUB = Rubella; ENT 2 = Complimentary strand of ENT1 of Enteroviruses; Row 4: Den1 = Dengue type1and 3 virus; Den2 = Dengue Type 2 and 4 virus and IC = Internal control.

Signature gene sequences chosen as probes for each of the pathogen were commercially synthesized (Metabion Inc., Germany). Specific probes for each of the pathogen (20 μM) were transferred on to a pre-determined position on the SES platform according to the templates. The SES platform comprised of a plastic frame mounted on a charged membrane on to which probes were arrayed at predetermined positions as indicated in Figures 1A,B. For each gene amplified, a single probe was used for hybridization except in case of HgD gene of HSV, MTR gene of CMV, and enteroviruses wherein an additional probe of the complementary strand was also used. Two different templates, one for identification of DNA pathogens and the other for identification of RNA pathogens (Figures 1A,B) were used for fixing of the probes onto the SES Platforms. The SES platforms were subsequently baked at 80°C for 20 min to immobilize the probes on to the platform. In order to monitor the amplification and the subsequent hybridization reactions internal controls were included in each run of the assay. For RNA pathogens ß-actin was used as an internal control while -globin was used as an internal control for DNA pathogens. The internal controls (IC) were always arrayed at a fixed position in the last row of each SES device (Figures 1A,B). This facilitated the correct orientation of the SES devise for reading the result of each hybridization reaction.

Detection of the amplified products was facilitated by using biotin labeled primers. Briefly, hybridization was carried out by heat denaturing the amplified product at 95°C for 10 min. The denatured amplified products were then diluted in the hybridization buffer and transferred on to the SES platform and the platform was incubated at 50°C for 30 min. Unbound amplicons were removed by washing the device thrice with pre-heated wash buffer. Following the washes, conjugate (Streptavidin peroxidase, Thermo, USA) diluted in conjugate buffer containing 1% BSA in PBS along with 0.05% tween 20 was added and incubated for 15 min at room temperature. Unbound conjugate was removed by washing thrice with the conjugate buffer at room temperature. Subsequently freshly prepared substrate (0.5 mgs/ml of Diaminobenzidine HCl with 0.03% of H_2_O_2_) was added and incubated for 10 min at room temperature. SES platforms were then washed with water and the signal observed with naked eye under adequate illumination. A semi-quantitative scale was developed in order to minimize inter—reader variability in interpretation of results. This was based on the intensity of the color of the signal visualized on the SES (Table 3).

**Table 3:** Depicting the signal intensity and its corresponding score.

**Sl. No**	**Signal intensity**	**Score**
1	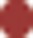	5 and above
2	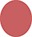	4
3	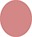	3
4	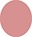	2
5	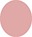	1
6	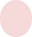	+/−

#### Standardization of the assay

The assay was standardized using the primers in uniplex format initially and subsequently was evaluated in a multiplex format. The standardization of the multiplex assay was a reiterative process wherein the concentration of various components used in the amplification were varied in order to obtain a comparable limit of detection in both uniplex and multiplex assay.

#### Specificity of the assay

The specificity of the primer and the probe set of each pathogen were ascertained by using nucleic acid obtained from all the standards. The nucleic acid extracted from all the standards were sequentially subjected to amplification with primers specific to the corresponding pathogen as well as primers of all other pathogens. Subsequently, all the amplified products were hybridized on a platform embedded with the specific probe corresponding to the primer set used for amplification. Furthermore, nucleic acid obtained from all the standards were sequentially amplified in a multiplex format and the amplified product was cross hybridized on to a platform embedded with all the other probes in order to ascertain the specificity of both amplification as well as hybridization. The concentration of the nucleic acid that was used in the specificity assay was 4 log dilutions above the determined limit of detection for that particular pathogen.

#### Limit of detection (LOD)/ analytical sensitivity

Serial dilutions of the standards (No. of organisms as measured plaque forming units or colony forming units or quantity of plasmid DNA) were subjected to nucleic acid extraction followed by amplification in both uniplex and multiplex format directly in case of DNA pathogens, whilst in case of RNA pathogens the LOD was determined by subjecting the viral standards to RNA extraction followed by cDNA conversion and amplification using a single specific set of primers initially in an uniplex format and subsequently in a multiplex format. The primer and probe set for a particular pathogen was finalized after carrying out a detailed specificity study as described above. Following this the analytical sensitivity of the assays were carried out using a series of experiments. Quantified DNA was serially diluted to test the analytical sensitivity initially in an uniplex format and later in the multiplex format. Wherever, a discrepancy was observed between uniplex and multiplex assays the amplification system was modified to attain an equivalent sensitivity. Once the analytical sensitivity was fixed the assay was carried out in triplicates to assess reproducibility of the assay. As a second step, quantified standards (either cultures/vaccines/ standards from QCMD) of each organism was serially diluted in negative CSF samples to establish the LOD in multiplex format. If the LOD varied from the analytical sensitivity, the amplification conditions were modified to attain an equivalent LOD. Once the LOD was fixed then, quantified standards equivalent to the established LOD was spiked in the negative CSF samples in triplicate and the whole assay from extraction to hybridization was carried out in triplicate to re-establish the LOD. As this test is intended only qualitative purposes, the presence of a clear visible 2+ intensity spot was considered to be positive. The amplification products were hybridized on to the SES platform.

#### Precision analysis

Precision of the assay was calculated after ascertaining the LOD in order to determine inter and intra assay variability of SES. Precision was determined by spiking two concentrations of the standards into a “*normal CSF”* (sample obtained from patients undergoing spinal anesthesia for minor surgery), one at the predetermined LOD and the other at double the concentration of the LOD. Multiple aliquots of the spiked standards were frozen at −70°C. Each day duplicates of each concentration were thawed and subjected to SES process—nucleic acid extraction to hybridization. The results of any particular analyte for all 10 days were scored by a single individual to ensure uniformity of the read out.

## Results

During the entire process of development of SES, if a given set of primers and probes were not sensitive enough to give LOD of clinical relevance at the stage of amplification and/or hybridization either in uniplex or in multiplex format, a new set of primers and probe were designed. Thus, the designing of primers and probes was a reiterative process.

**Figure 2:**
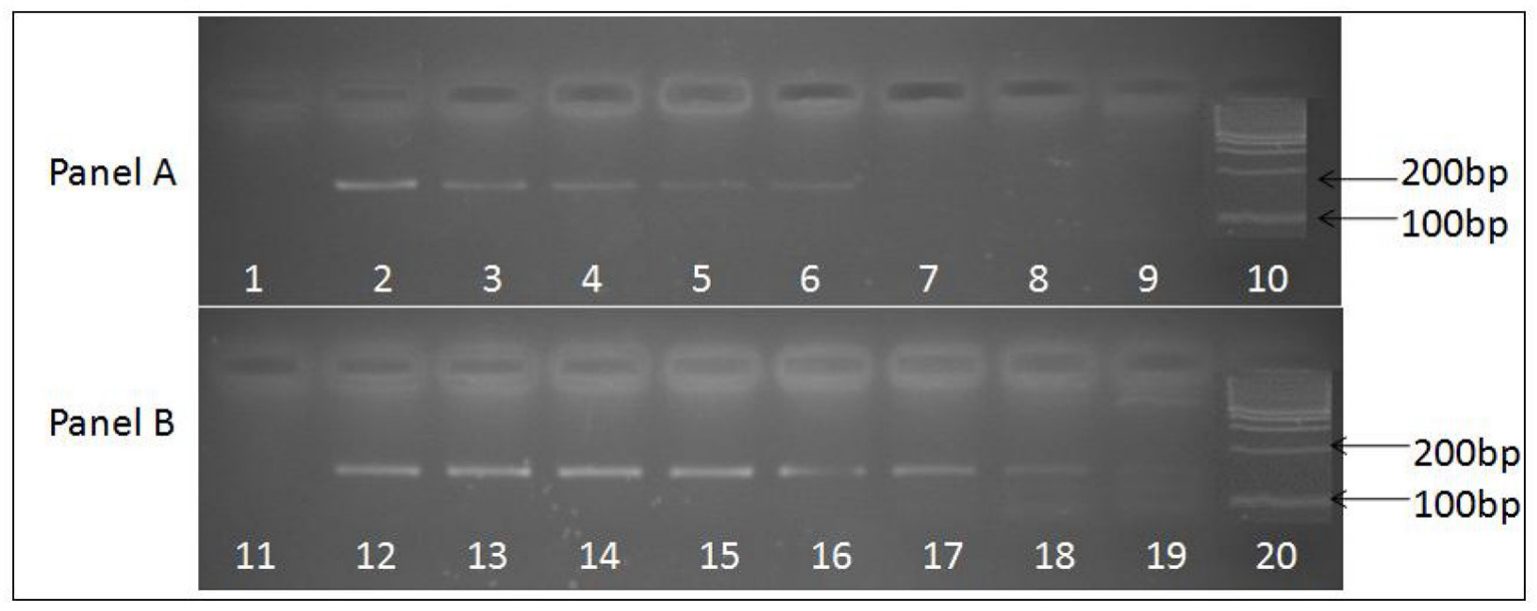
Ethidium bromide stained agarose gel image depicting 156 base pair uniplex PCR amplified product obtained from JE viral cDNA prepared by two methods. **(A)** Depicts PCR amplified products obtained using cDNA prepared with random primers, while **(B)** depicts PCR amplified products obtained using cDNA prepared with JEV specific primers. Serially log dilutions of cell culture grown JE virus ranging from10^6^ to 0.1 particle/ml was used to extract RNA and preparation cDNA. Lanes 1 and 11, depict results obtained with Negative control, Lanes 10 and 20, represents 100 bp molecular weight markers (DNA ladder), Lanes 2 to 9 and 12 to 19 represent the PCR amplified product of log dilutions of JEV 10^6^ particles/ ml to 0.1 JEV particles/ml respectively. The PCR sensitivity obtained with cDNA prepared using specific primers (lane 19, 0.1 particles/ml) is higher than the cDNA prepared using random primers (lane 6, 10^2^ particles/ml).

**Figure 3:**
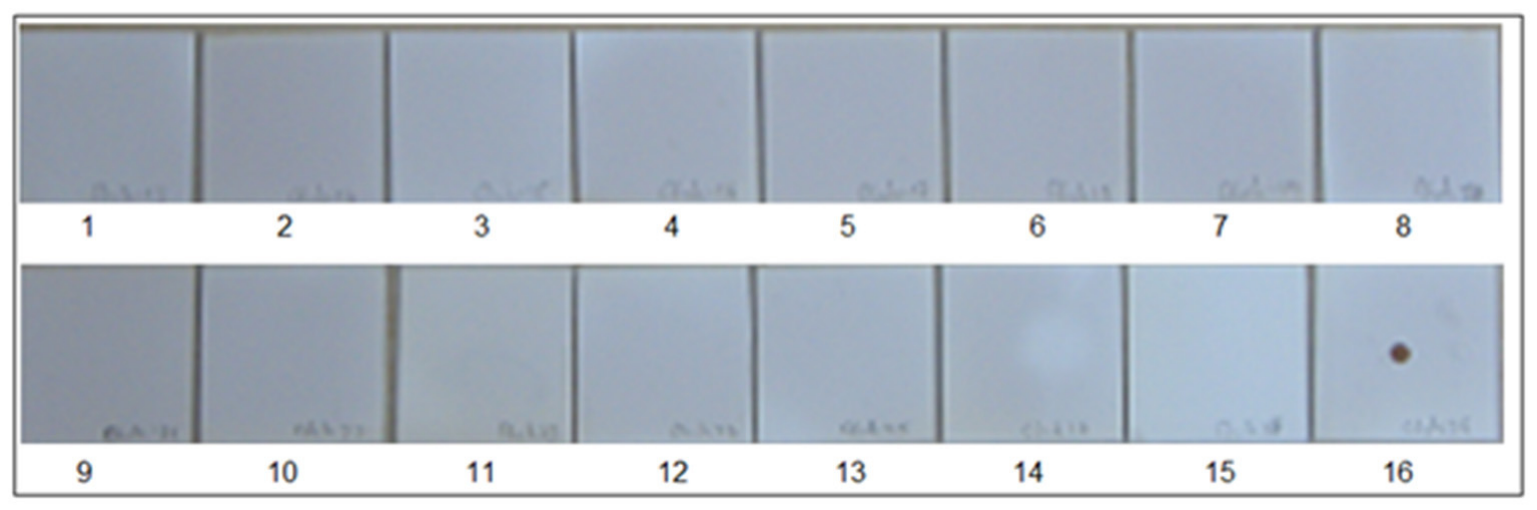
Specificity of Chikungunya primer and probes set in SES. cDNAs were prepared for various RNA viruses as described in Materials and Methods and subjected to amplification and hybridization using Chikungunya primer and probe sets. Each panel depicts results obtained thereof. Panel 1 Depicts results obtained with negative control while Panel 16 depicts results obtained with Positive Control i.e., Chikungunya cDNA. Panels 2–15 depict results obtained with Dengue 2(Panel 2), JEV (Panel 3), West-Nile(Panel 4), Polio(Panel 5), Coxsackie B1(Panel 6), Coxsackie B2(Panel 7), Coxsackie B3(Panel 8), Coxsackie B4(Panel 9), Measles(Panel 10), Mumps(Panel 11), Rubella(Panel 12), Rabies(Panel 13), Nipah(Panel 14), and Chandipura (Panel 15) viruses respectively. Lack of signals in Panels 2 to 15 indicates that no cross amplification and/or hybridization was noted with cDNA's/ plasmid DNA of all RNA viruses, thereby indicating specificity in the RNA SES for Chikungunya primer and probe set.

**Figure 4:**
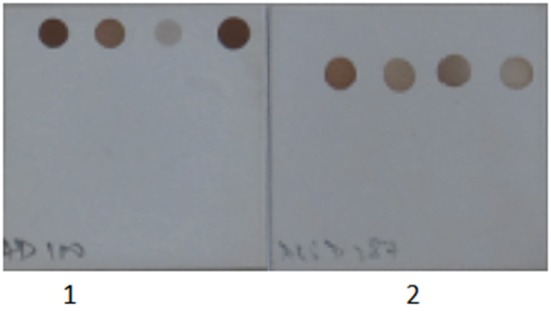
Specificity of Herpes Simplex Virus and Cytomegalovirus primer and probes set used in the SES. 1. Herpes Simplex Virus DNA was amplified in multiplex format and hybridized on a SES platform. 2. Cytomegalovirus DNA was amplified in multiplex format and hybridized on a SES platform. Only signals specific to the organism identified is observed indicating no cross reactivity with genomically identical virus.

Primers and probes used in the SES were designed and synthesized for eleven DNA and eleven RNA pathogens. Primer and probe sets for all the pathogens were designed using the conserved sequences within the virulent genes. All primers were designed such that they could be used in a multiplex amplification system with universal cycling condition. The primers were initially evaluated for their ability to amplify the nucleic acid obtained from the standards for which they were designed in an uniplex format using agarose gel electrophoresis as the end read out (data not shown). In case of RNA viruses an additional step of cDNA conversion is involved and hence cDNA's prepared using specific primers were used as this cDNA gave a better sensitivity than the cDNA prepared using random primers (Figure 2). Suitability of the probes and the concentration of the probes were standardized by hybridization of the amplified products. It was noted that all the primers and the probes designed were specific for the pathogens for which they were designed. Specificity of all primer and probe sets were evaluated in the SES by using one set of primers and probes for amplifying and hybridizing the DNA's or cDNA's obtained from all pathogens. A representative example of the results obtained with Chikungunya primer probe set is depicted in Figure 3. As evident from the Figure 3, a positive result was obtained only with the primer and probe set specific to Chikungunya virus. Note that no signal was observed with any of cDNA's of other RNA viruses tested in the SES indicating specificity of Chikungunya primer and probe set. Nucleic acid obtained from DNA pathogens were also subjected to amplification and hybridization using Chikungunya primers and no cross reactivity was observed (Data not shown). Similar experiments were carried out for all the DNA and RNA pathogens (Data not shown). Further, the specificity of the probes used was ascertained by amplifying each pathogen and hybridizing the product on the SES platform embedded with all the probes. An example of the results obtained with HSV and CMV DNA is depicted in Figure 4. As evident from the figure, the positive signals were observed only with probes specific to HSV and CMV clearly indicating that the amplified HSV or CMV DNA did not cross hybridize with their genomically related pathogens.

**Figure 5:**
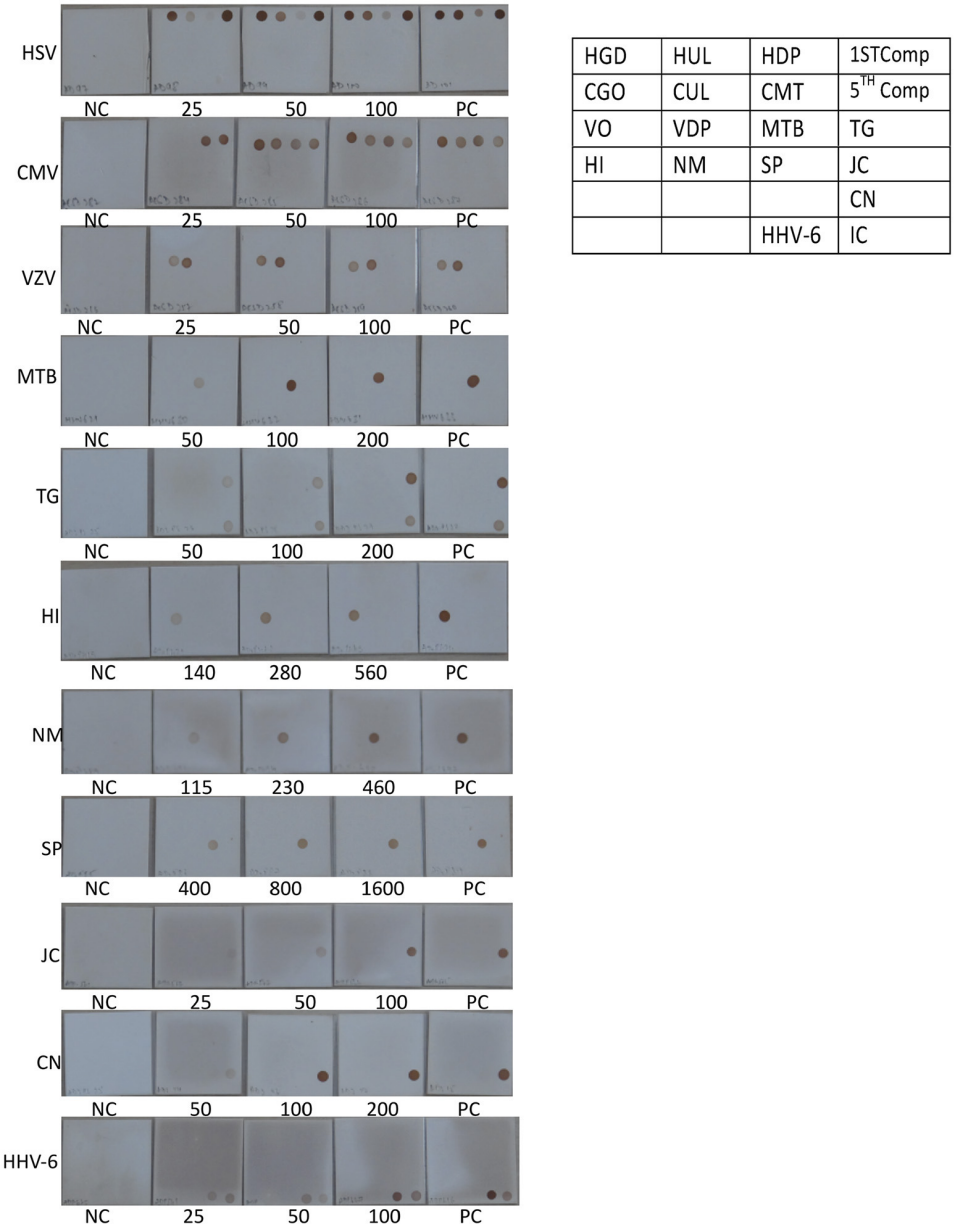
Determination of Limit of detection (LOD) for DNA pathogen on the SES platform. DNA extracted from defined concentrations of viruses/bacteria /fungi/parasites were subjected to multiplex PCR and hybridization on the SES platform (Vide Materials and Methods for details). As indicated in the template provided on the right, multiple genes were amplified and hybridized with respective probes for HSV (row 1 of template), CMV (row 2 of template),VZV (row 3 of the template), and single genes were amplified and hybridized in case of *M. tuberculosis* and *T. gondii* (row 3 of the template), *H influenza, N. meningitidis, S. pneumoniae* and JCV (row 4 of template), *C. neoformans* (row 5 of the template), and HHV-6 and Internal control (row6 of the template). Each panel depicts NC = negative control. PC = Positive Control and the numbers written below each SES indicate the number of particles/CFU/organisms/ml that has been used for DNA extraction, amplification and hybridization in a multiplex format. (refer to Table 4).

**Figure 6:**
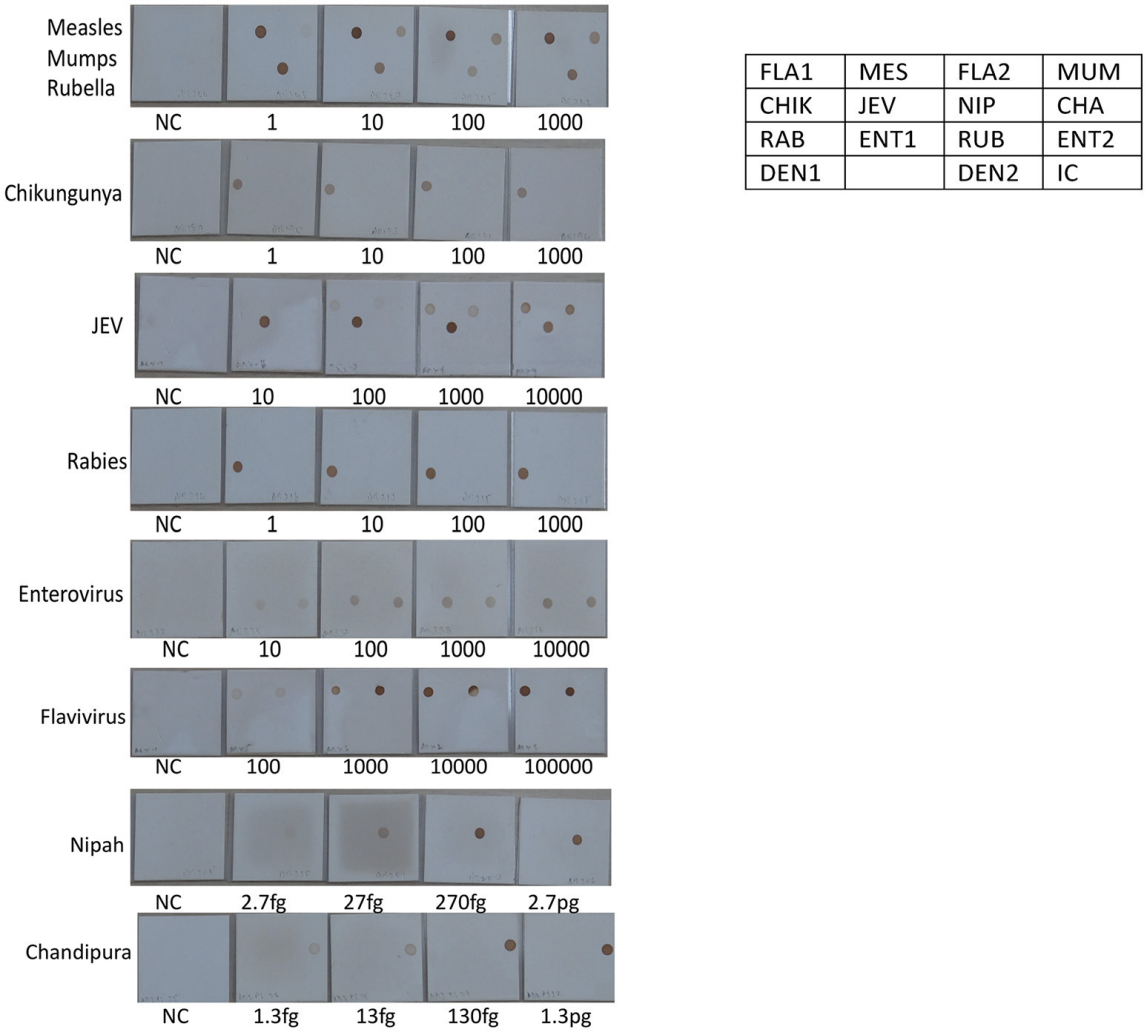
Determination of Limit of detection (LOD) for RNA viruses on the SES platform. RNA extracted from defined concentrations of viruses was subjected to, cDNA conversion, multiplex amplification and hybridization on the SES platform (Vide Materials and Methods for details). As indicated in the template provided on the right side, the Limit of Detection obtained in the SES for various RNA viruses in the various panels above. NC = Negative control and the numbers written below each SES indicate the number of viral particles/ml that has been used for RNA extraction, amplification and hybridization in a multiplex format. The limit of detection for Measles, Mumps, Rubella, Rabies, Entero and Chikungunya viruses was 1 particle /ml. The limit of detection for JEV using panflavivirus primers was 100 particles/ml whilst it was 10 particle/ml when JEV specific primers were used. The limit of detection for Nipah and Chandipura plasmids was 2.7 fg and 1.3 fg respectively.

The next step in the development of SES involved the determination of Limit of Detection (LOD) of the assay in multiplex format using the quantified pathogen standards diluted in “*normal CSF.”* As evident from Table 4 the LOD was 25 particles/ml with respect to all DNA viruses except VZV (50 particles/ml) and in case of bacteria it ranged between 50 cfu/ml in case of *M. tuberculosis* to 400 cfu/ml of *S. pneumonia* (Figure 5). Similarly, the LOD was 1 pfu/ml for all RNA viruses except in case of JEV and Enteroviruses which was 10 pfu/ml in the multiplex format. In case of Nipah, Chandipura, and Dengue viruses the LOD could not be determined as these virus standards with known titers were not available. Hence, the LOD could be expressed in femtograms of plasmid DNA/ml for Nipah and Chandipura viruses which were obtained as recombinant plasmids in to which “N” gene of these two viruses were cloned individually. Similarly, in case of *C. neoformans*, the LOD was determined using quantified nucleic acid extracted from the culture. This was done as there are no standard protocols for the determination of particle count of *C. neoformans* or other capsular fungi. As part of standardization of the assay, LOD was determined in both uniplex, and multiplex format. Furthermore, in case where the LOD varied between uniplex and multiplex assay, the assay was tuned so as to obtain the same LOD in both format. However, this could not be achieved in case of two RNA viruses, JEV, and Enteroviruses, wherein the LOD in uniplex format was 1 pfu/ml when compared to multiplex it is 10 pfu/ml. Furthermore, during the determination of LOD for Measles, Mumps and Rubella it was noticed that SES detected all the three pathogens simultaneously at their LOD levels (Figure 6). This was further established by comparing the LOD for Mumps virus using both Mumps vaccine and the MMR vaccine. The ability of SES in detecting multiple pathogens simultaneously was further established by using CSF samples which were spiked with two different pathogens at their LOD levels or one at the LOD and the 10 times higher concentration than the LOD. The results obtained clearly indicate that the SES is not only capable of detecting single organism but also polymicrobials if present in a clinical sample.

**Table 4:** Comparative LOD for all the pathogens obtained in uniplex and multiplex assay formats of the SES.

**Organism**	**Limit of detection (organisms pfu or cfu/ml)**	
	**Uniplex**	**Multiplex**
HSV	25	25
HHV-6	25	25
CMV	25	25
VZV	50	50
JC	25	25
*M. tuberculosis*	50	50
*T. gondii*	50	50
C.neoformans^*^	50	50
*S. pneumoniae*	400	400
*H. influenzae*	140	140
*N. meningitidis*	115	115
JEV	1	10
Measles	1	1
Rubella	1	1
Mumps	1	1
Chikungunya	1	1
Rabies	1	1
Nipah	2.7fg	2.7fg
Chandipura	1.3 fg	1.3 fg
Dengue	Not determined	Not determined
Polio	1	10

*Except for JEV and Enteroviruses (Polio) the LOD is uniform in both uniplex and multiplex assay formats. However, the LOD obtained for these two viruses in the multiplex assay format was well within the reported clinical sensitivity range including JEV and Enterovirus. Sensitivity for Dengue virus is not determined due to the unavailability of titered standard*.

* *In case of C. neoformans the LOD was derived based on the quantity of DNA that was detected in a multiplex assay*.

**Figure 7:**
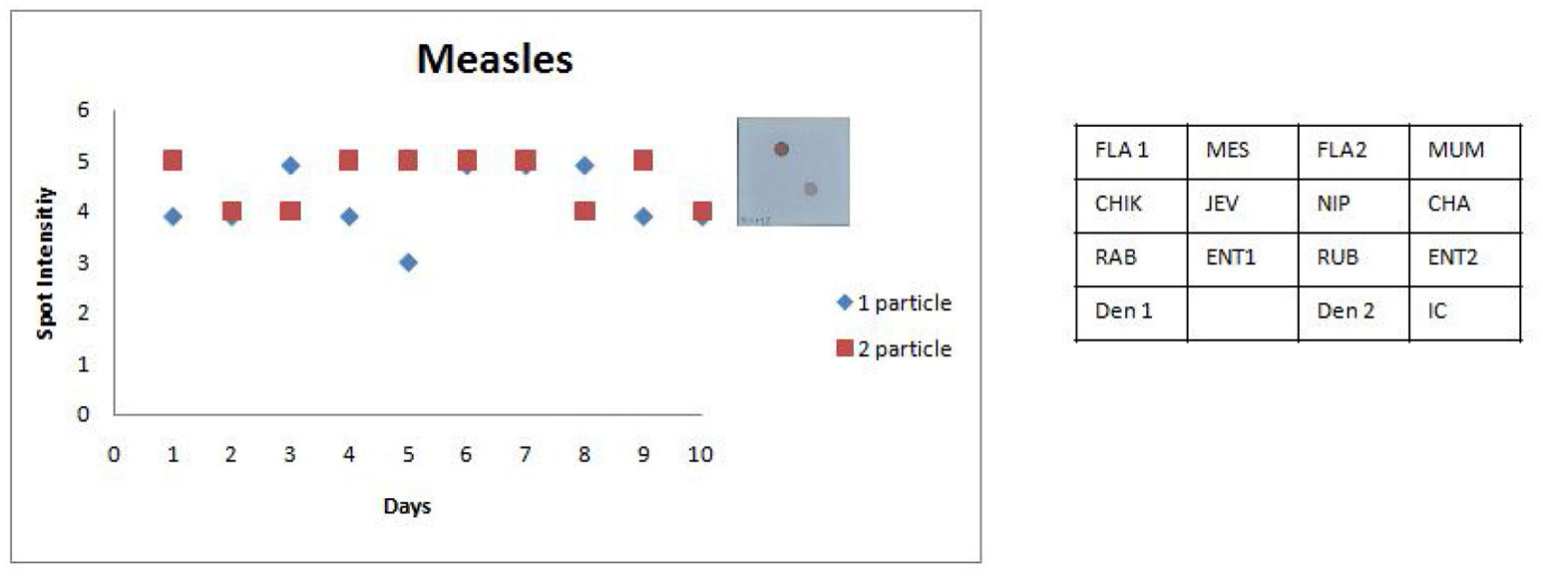
Scatter plot depicting the results of the Standard Precision assay for Measles virus. X axis indicates the number of days, Y axis indicates the average spot intensity scores obtained for each of the two concentrations of template used; blue = concentration of template at LOD and red = concentration of template double that of LOD. A representative hybridization image depicting a score of 5 on the spot intensity scale obtained with twice the LOD of Measles Virus is depicted adjacent to the graph. As evident from the graph there is no significant inter or intra assay variability obtained in the SES for Measles Virus over a ten day period.

**Figure 8:**
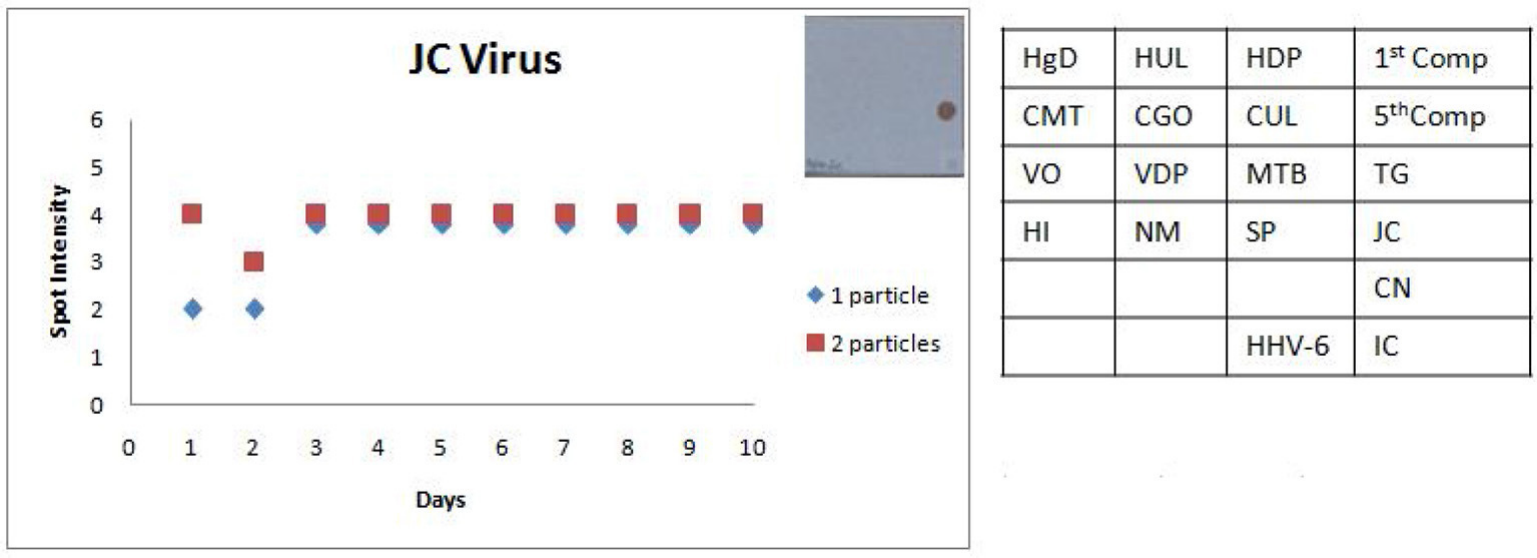
Scatter plot depicting the results of the Standard Precision assay for JC virus. X axis indicates the number of days, Y axis indicates the average spot intensity scores obtained for each of the two concentrations of template used; blue = concentration of template at LOD and red = concentration of template double that of LOD. A representative hybridization image depicting a score of 4 on the spot intensity scale obtained with the LOD of JC Virus is depicted adjacent to the graph. As evident from the graph there is no significant inter or intra assay variability obtained in the SES for JCVirus over a tenday period.

The last step in the development of SES was followed by the assessment of the reproducibility of the assay by performing the precision studies for each of the pathogen except for Chandipura virus, Nipah virus wherein plasmid constructs were used as standards and in case of Dengue virus where in a quantified virus standard was not available. As per the Clinical Laboratory Improvement Amendment (CLIA) standards, the precision assay for all the pathogens was carried out by spiking the CSF with two different concentrations one the LOD and the other double the concentration of the LOD. Precision assay for *C. neoformans* was carried out using quantified nucleic acid extracted from the culture, due to the non-availability of a standard quantification protocol to determine the number of organisms. Standards obtained from QCMD/vaccine/culture were spiked into “*normal CSF*” obtained from healthy controls undergoing spinal anesthesia for minor surgeries. A representative scatter plot for one RNA and one DNA pathogen is presented in Figures 7, 8. All the precision assays were indeed multiplex amplification reactions. It can be observed from these figures that SES did not exhibit any significant inter assay variation monitored on ten different days or any intra assay variations observed between the two aliquots of each concentration on any given day. Further it indicates that SES developed is highly robust and ready to be used clinically after a clinical validation.

## Discussion

For several decades in India, JE has traditionally been considered as the only major cause of AES. Recent data from the NVBDCP in the past decade as well as several other reports have indicated that JEV accounts merely for 10–12% of all reported AES cases especially after the phased introduction of JE vaccination in India since 2006. Other etiologies, including enteroviruses, and Nipah Virus have also been implicated. However, there have been no systematic efforts on a large scale to investigate the etiologies other than JEV (14).

Most dramatic improvement of outcomes in the management of encephalitis can be brought about by early and accurate detection of the aetiological agent. Epidemiological and clinical parameters though helpful in arriving at the diagnosis of pathogen in a small percentage of cases, are indistinguishable in a majority of patients (15). Algorithm based sequential performance of available repertoire of all laboratory tests is cumbersome, costly, and time-consuming negating the very purpose of the whole exercise. In most of the clinical settings, CSF analysis of cells, protein, and glucose are used to arrive at diagnosis of viral or bacterial infections. This analysis seldom yields definitive diagnosis and thus has not been very useful clinically. Most of the AES cases are treated with anti-virals such as acyclovir and antibiotics. In order to improve the speed and precision of diagnosis as well as to provide an evidence-based rationale for starting treatment, a SES was developed. Indeed this assay was capable of simultaneous detection of 22 different pathogens known to cause AES in India (16–31).

Multiplex amplifications reported hitherto have aimed at diagnosis of all viruses from a single family such as enteroviruses and herpes viruses (7, 32). Real Time PCR based tests could multiplex up to 3 to 5 (33–35) different pathogens while fluorescent detection in combination with melting temperatures such as SeptiFast or Fast-track or TLDA cards or Biomerieux-diagnostics filmarray-meningitis-encephalitis-ME-panel can radically enhance the multiplexing ability. However, it requires multiple extractions of CSF to be distributed across multiple reaction panels. This is generally not feasible in the Indian context as most of these panels do not encompass the AES pathogens relevant to the Indian context. Furthermore, in pediatric patients where the amount of sample available is limited this poses a serious limitation. With the advent of recent technology like Micro-array (36), diagnostic capabilities have improved in research laboratories. Although, this system allows for possibility of multiplexing clinical relevance is limited as the end read out in the form of signal to noise ratio of all genes tested (some geographically irrelevant) renders interpretation of the results ambiguous. Microarrays are costly and require sophisticated infrastructure. Consequently, there is an urgent need to develop a diagnostic tool for the simultaneous detection of all the probable pathogens causing AES especially for developing countries. SES involves multiplex amplification of select set of specific genes for all probable pathogens and identification of the amplified product by hybridization. Moreover, the generic thermal cycler, generic thermal incubators, inexpensive hybridization device—SES platform developed specifically for this purpose and the visual detection of the colored signal make this test widely applicable across many moderately equipped clinical laboratories.

The careful selection of primer and probes used in amplification and hybridization has not yielded any spurious products. Furthermore, the designed primer sets have not amplified genes of the pathogens other than the intended ones. They have neither interfered with hybridization nor resulted in cross hybridization. We believe that this is one of the crucial factors that contributed to the success in the development of the SES. We observed that the cDNA prepared using random primers did not given an adequate sensitivity. This we believe is a combined effect of excess RNA contributed by the human inflammatory cells in the CSF and the rapid degradation of the viral RNA. However, we noted that cDNA prepared using multiplex specific primers yielded adequate sensitivity for the detection of all RNA viruses.

The LOD of most of the RNA pathogens were within the range of 1–10 pfu/ml while that of DNA pathogens was between 25–400 pfu or virions/ml. These concentrations are sufficiently low to be clinically significant (5). There are only few reports wherein the RNA pathogen load with respect to the infection was measured (37). The LOD reported in our study for mumps virus and enteroviruses (37) is far below the concentrations required to cause an infection. Hence, we extrapolate that the LOD of SES for all the pathogens is sufficiently low to detect all cases of encephalitis early during the infections. Simultaneous detection of Measles, Mumps, and Rubella by SES when MMR vaccine was used clearly demonstrated that SES was capable of detecting multiple pathogens. This was further proved by experiments carried out by spiking multiple pathogens into a single CSF sample.

The SES described here has been standardized and fine-tuned to minimize inter and intra assay variability. The results of the precision study (Figures 6, 7) indicate the following features; (i) the LOD was not compromised when the assay was performed by different technicians on ten different days and (ii) minor variations in signal intensity if any, were well within acceptable limits and did not affect the final result of the test. In conclusion, the results of this study unambiguously demonstrate that the SES system has adequate analytical sensitivity, specificity and rapid turnaround (7 h) time rendering it suitable for clinical application after validation. The results of such a comprehensive clinical validation forms a part of a peer reviewed separate investigation.

## Author contributions

SG, LL, RB, and RV contributed in conception and design of the development. SG and SA designed the primers and probes required for the development of SES. SG wrote the whole manuscript which was read, revised, and approved by RB and RV.

### Conflict of interest statement

The authors declare that the research was conducted in the absence of any commercial or financial relationships that could be construed as a potential conflict of interest.
